# 

**Published:** 1891-02

**Authors:** 


*Texas State Medical Association—Section on Practice of Medicine.*

It is the earnest desire of the chairman that this Section shall be well represented by contributions at the meeting in Waco next April. The active co-operation of all members interested in practical medicine, and in promoting the usefulness of the Association, is hereby cordially invited.

Attention is called to a resolution adopted at the last meeting (see page 306 of the Transactions), insisting upon the observance of the by-laws requiring papers, or their *titles*, to be furnished to secretaries of sections at least a month before the meeting which is to act upon them.

The discussion of papers, as appearing in our Transactions, have been notoriously deficient for the reason that members not knowing the titles of papers could not prepare themselves, and the secretary, without assistance, could not make an accurate or full report. It was to correct these well known evils that the Association adopted the above mentioned resolution.

Members who propose to read papers before this Section will please see that the *titles* of their papers shall be in the hands of Dr. B. F. Church, Terrell, at as early a day as is possible. Dr. Church will attend to their prompt publication.

H. A. WEST, M. D., Chairman, Galveston.
B. F. CHURCH, M. D., Secretary, Terrell.

